# A comparison of two methods for T cell epitope mapping: “cell free” in vitro versus immunoinformatics

**DOI:** 10.4172/1745-7580.1000045

**Published:** 2011-05

**Authors:** Timothy J. Messitt, Frances Terry, Leonard Moise, William Martin, Anne S. De Groot

**Affiliations:** 1 EpiVax, Inc., Providence, RI, USA; 2 Institute for Immunology and Informatics, University of Rhode Island, Providence, RI, USA; 3 The Warren Alpert Medical School of Brown University, Providence, RI, USA

## Abstract

**Background:**

Methods for identifying physiologically relevant T-cell epitopes are critically important for development of vaccines and the design of therapeutic proteins. As the number of proteins that are being evaluated for putative immunogenicity expands, rapid and accurate tools are in great demand. Several methods to identify T-cell epitopes have been developed, the most recent of which is a cell free system consisting of a minimal set of proteases incubated with HLA DRB1*0101, HLA-DM and whole antigen. Isolation and sequencing of the HLA bound peptides using mass spectrometry allows for the prospective identification of immunodominant T-cell epitopes.

**Results:**

We present here, a comparison of this cell free in vitro antigen processing system to an immunoinformatics approach using the EpiMatrix algorithm. Our comparison reveals that in addition to identifying a similar set of epitopes to the cell-free system, the immunoinformatics approach prospectively identifies more HLA-DRB1*0101 epitopes and can simultaneously analyze multiple HLA alleles.

**Conclusions:**

Although the cell-free system incorporates antigen processing and MHC binding, the immunoinformatics approach identifies many validated epitopes with a very high degree of accuracy and can be performed much faster with far fewer resources.

## Background

Methods for the prospective identification of physiologically relevant T-cell epitopes are critically important for development of vaccines and for the design of therapeutic proteins. A cell free system (CFS) for prospectively identifying T-cell epitopes from whole antigens was recently described and applied to the identification of influenza epitopes [1]. As described by Hartman et al. in their publication, CFS epitope mapping was performed by pre-incubating whole antigens with HLA-DRB1*0101 and HLA-DM, and then exposing the mixture of antigen and HLA DR/DM to a minimal set of proteases, followed by isolation and sequencing of the HLA-bound peptides using mass spectrometry. The CFS was initially validated using two model antigens (HA1 from influenza A/Texas/1/77 and type II collagen) as positive controls and then applied prospectively for the discovery of new HLA-DRB1*0101 immunodominant epitopes from a recombinant liver-stage antigen of malaria falciparum (LSA-NRC) and HA1 from H5N1 influenza (Viet Nam).

The publication of the CFS method provided an opportunity for comparing a purely immunoinformatics approach based entirely on MHC binding affinity (EpiMatrix) to an in vitro system that involves both antigen processing and presentation [2]. We hypothesized that predicted MHC binding (as performed in silico) would provide results that were at least equivalent to the more laborious CFS approach. As the identification of T cell epitopes using the CFS approach. requires a significant amount of laboratory effort, reagents, and specific expertise in the use of MALDI mass-spectrometry, the immunoinformatics approach might, in addition, offer significant time and cost savings. As is described here, our detailed comparison reveals that the immunoinformatics method correctly identified four of the six epitopes identified by the CFS method, at lower cost and with greater time efficiency, and, in addition, identified other potential epitopes that appear to have been missed by the CFS. Neither of the two CFS epitopes that were missed by EpiMatrix were validated in follow up assays. In the brief report below, we provide a detailed comparison of the *in silico* approach using EpiMatrix and the CFS approach, in terms of epitopes identified and the relative speed, effort required and cost of the two methods.

## Results

### CFS reductionist method

The CFS approach to finding immunodominant epitopes, as published in reference 1, is described here for comparison with the EpiMatrix method. The cell free system (CFS) is restricted to evaluations of a single HLA at a time. The assay requires combining a minimal set of components for antigen processing (full length antigen, human MHC Class II HLA-DRB1*0101, HLA-DM, and Cathepsins S, B, and H) under both endosomal and lysosomal conditions. Hartman et al. describe the application of the CFS method to four proteins: (1) an artificial construct of influenza H1N1 (A/PR/8/34) HA with a single, well-known DR1-restricted epitope (A/Texas/1/77 HA_306–318_) appended to the C-terminus; (2) Collagen type II; (3) influenza H5N1 (A/Vietnam/1203/2004) HA and (4) Liver stage malaria antigen. The resulting peptide-DR1 complexes were isolated by immunoprecipitation and the bound peptides were eluted under acidic conditions. These eluted peptides were then analyzed on a matrix-assisted laser desorption ionization (MALDI) mass spectrometer.

Results for the CFS method were obtained using a single allele (HLA-DRB1*0101) [1]. The eluted epitopes were validated in vitro using T cell proliferation, cytokine induction, tetramer staining, or some combination of the three following immunization of HLA-DRB1*0101 mice with the whole protein antigen.

For example, recombinant HA1 (rHA1), engineered to include a published epitope, was incubated in the cell free system. After isolating HLA DRB1*0101 complexes, the genetically-linked A/Texas/1/77 known immunodominant epitope and only one other peptide (A/PR/8/34 HA_298–317_) were eluted from peptide-DR1 complexes. T cell proliferation assays using peripheral blood mononuclear cells (PBMC) obtained from HLA DRB*0101 transgenic mice immunized with rHA1 showed a strong dose dependent response to the A/Texas/1/77 single epitope identified by the cell free assay and a weaker but still significant response to the A/PR/8/34 peptide. The sum of proliferative responses (ex vivo) to these two epitopes approached the magnitude of the response to whole rHA1.

As a second example, bovine type II collagen (CII) was used to test the epitope identification system. CII is a major component of cartilage and is the main suspected auto-antigen in rheumatoid arthritis in DR1+ individuals [4]. A core DR1 restricted immunodominant epitope, CII_282–289_, has been identified in CII in mouse studies. Following enzymatic digestion and incubation in the CFS, one peptide was eluted, CII_273–305_, as well as variants of that peptide that share the same core epitope. Proliferation studies performed with T cells from CII-immunized mice validated the eluted CII_273–305_ epitope.

**Figure 1:**
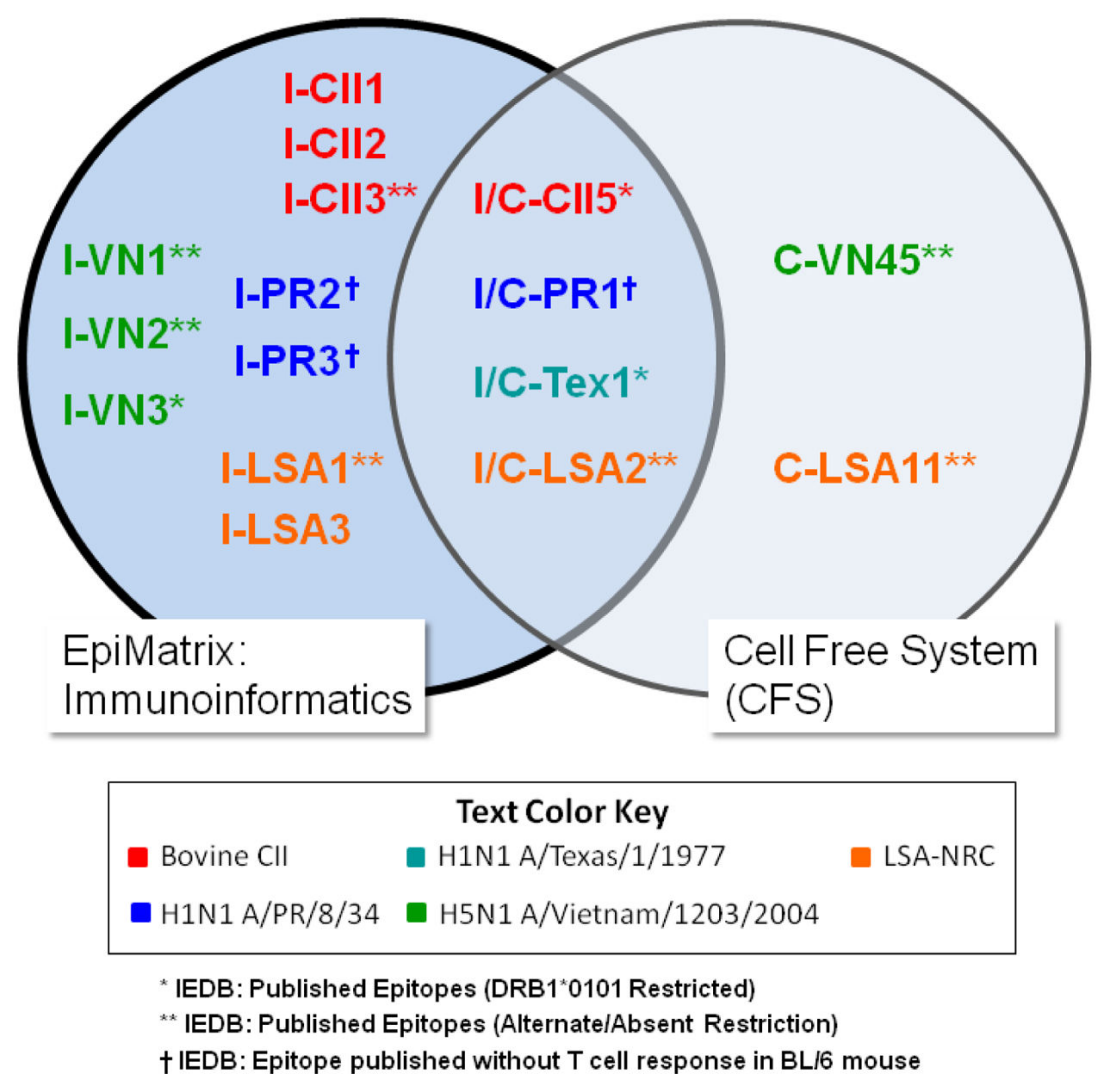
EpiMatrix prospectively identifies more potential epitopes than the CFS Epitopes identified by the CFS are labelled C, epitopes discovered by Immunoinformatics are labelled I and epitopes discovered by both are labelled I/C; the rank in the HLA DR1 EpiMatrix analysis for the antigen is indicated by the number 1–5; note that only one epitope from influenza A Texas was used (recombinantly fused to the PR protein) rather than the whole protein and therefore this single epitope is given the first rank (I/C-Tex1).

The CFS was also used to prospectively identify immunodominant DR1 epitopes from HA1 protein of influenza A/Vietnam/1203/2004 (H5N1) and LSA-NRC, a recombinant modified version of a protein expressed exclusively in malaria-infected hepatocytes at a preerythrocytic stage, which was designed as a vaccine against preerythrocytic stage malaria. Several new epitopes (Figure 1) were identified in these previously unmapped proteins and subsequently validated in T cell assays following immunization of HLA-DR1 transgenic mice with the whole antigen in CFA.

### Immunoinformatics method

The sequences of the four antigens evaluated in the CFS were obtained from GenBank and then analyzed using EpiMatrix [EpiVax, Providence USA]. Standard criteria (EpiMatrix score in top 5% of scores on a Z scale) were used for epitope identification [2]. A list of the EpiMatrix-predicted HLA DRB1*0101 epitopes was created and compared to the epitopes identified by the CFS. EpiMatrix prospectively identified four of the six epitopes that were identified by the CFS (67%), and one epitope from each of the antigens; I/C-CII3, I/C-PR1, I/C-Tex1, and I/C-LSA2 (Table 1). These epitopes are shown at the intersection of the Venn diagram in Figure 1 and labelled I/C to denote that they were identified by both the Immunoinformatics (I) and the CFS (C) approaches.

All four I/C epitopes scored among the top 5 HLA DRB1*0101 EpiMatrix scores for the antigens (the EpiMatrix rank is indicated by the numerical suffix in Table 1 and Figure 1). Both of the “C” epitopes, which were discovered by the CFS but not by EpiMatrix, scored within the top 10% of EpiMatrix scores, which is below the top 5% cut-off that would normally be used for the selection of T cell epitopes by EpiMatrix analysis. We note that one of these epitopes, selected by the CFS (C-VN45) has been reported to be HLA DRB1*0401- and HLA DRB1*1101-restricted according to IEDB, but not HLA DRB1*0101-restricted. Consistent with the IEDB-reported findings, DRB1*0401 and DRB1*1101 EpiMatrix scores for this epitope are in the top 1% of predicted binders. The second epitope identified by the CFS and not by EpiMatrix (C-LSA11) fell just below the EpiMatrix cut-off for a positive score (1.58, rather than 1.64, see Methods).

As compared to the CFS, the EpiMatrix immunoinformatics approach identified many more prospective epitopes for each of the antigens. Performing the analysis as described here, a total of 13 epitopes were identified based on their HLA DRB1*0101 score: these included the three highest-scoring epitopes for each of four antigens that were evaluated by the CFS and the one high-scoring HLA DRB1*0101 epitope from A/Texas (only one published epitope was included in the rHA protein tested in the CFS). Of these 13 epitopes, eight (62%) were previously confirmed as human T cell epitopes, according to IEDB; validation is not yet recorded the remaining five epitopes.

### Detailed comparison of CFS and EpiMatrix results

Epitope sequences identified by both the CFS and EpiMatrix method were cross-referenced against the Immune Epitope Database (IEDB) of published T cell epitopes and MHC ligands using the substring parameter. Further, all T cell responses and MHC binding results available on IEDB were compiled for each input antigen. If IEDB is taken as the reference standard for validation of epitopes predicted by either method, EpiMatrix prospectively identified five more ‘validated’ epitopes that the CFS method did not identify. CFS identified two epitopes that were not identified by EpiMatrix, and although these are published in IEDB, they have been published for alternative alleles. If more stringent criteria are applied, (restriction by HLA DRB1*0101 and publication in IEDB), EpiMatrix correctly identified three of the three (100%) HLA DRB1*0101-restricted epitopes. This significant reduction is due in part to the limited HLA-restriction references available on IEDB; of the four test antigens, only H5N1 Vietnam had references qualified as HLA-DRB1*0101-restricted. As we will discuss in greater detail below, the single HLA DRB1*0101-restricted epitope prospectively identified by the CFS (I/C-CII5), was only validated as a result of querying the EpiMatrix-identified core.

#### Influenza PR antigen

The single PR epitope that was identified by both the CFS and EpiMatrix, I/C-PR1, was ranked first of all PR-derived HLA DRB1*0101 epitopes by EpiMatrix, and was extracted from the same amino acid locus (306–318) as the influenza HA epitope control peptide (I/C-Tex1) that was fused to the A/PR/8/34 antigen. The two epitopes also identified by EpiMatrix have high DRB1*0101 scores (I/C-PR1 and I/C-Tex1) and contain an epitope bar (or EpiBar) [6,7], a feature that is often present in promiscuous epitopes [5], and that has been associated with immunogenicity in human studies [8,9]. This important feature of promiscuous, immunogenic epitopes is not detectable using the CFS. EpiMatrix also identified two additional epitopes in influenza A/PR/8/34 HA (Table 1).

#### Influenza A Viet Nam

When the full length HA1 from H5N1 A/Vietnam/1203/2004 was analyzed in the CFS, two unique peptide species were identified; both shared the same core sequence (C-VN45). The authors selected the shorter of these two peptides (HA_259–274_) to test for immunodominance in T cell proliferation assays, cytokine production assays, and tetramer staining assays. EpiMatrix analysis shows that the shorter HA most likely has a truncated terminal HLA DRB1*0101 HLA binding motif. The peptide only has one EpiBar at the C-terminal end of the sequence and the N-terminal end of the peptide, which is devoid of predicted HLA binding motifs, would interfere with binding, *in vitro*, and with immunogenicity. The properly centered peptide would have been identified prospectively using EpiMatrix.

Three other epitopes were predicted by EpiMatrix in the same protein to be better HLA DRB1*0101 binders, but they were not identified by the CFS. All three have been experimentally validated and published; only one was confirmed for HLA DRB1*0101. Based on the fact that the third-ranked EpiMatrix epitope is published and found to be an HLA DRB1*0101 epitope in IEDB, the other two (ranked 1 and 2) are equally likely if not more likely to be HLA DRB1*0101-restricted, however this would have to be tested prospectively in T cell assays as was done for the CFS epitopes.

#### Collagen

It is notable that the single collagen epitope (I/C-CII5) identified by the CFS was also confirmed by EpiMatrix. It was the 5th ranked peptide for HLA DRB1*0101, which is not unexpected considering the greater length of the CII sequence compared to other proteins examined in the CFS. The peptide has a maximum DR1 EpiMatrix score of 2.66, putting it in the top 1% of peptides expected to bind to DR1, a rank that would have normally led to selection by EpiMatrix. The sequence of the CFS eluted peptide CII_273–305_ contains the same epitope that is predicted by EpiMatrix, however its sequence is elongated and the probable HLA binding motif is almost obscured by the extended flanks that were eluted with the core sequence. When queried against the IEDB, the CFS-eluted sequence returned no results, whereas the EpiMatrix-identified core sequence was published as a DRB1*0101-restriced epitope. In other words, immunoinformatics analysis using EpiMatrix can rapidly and accurately identify a core HLA binding epitope, whereas the CFS does not. EpiMatrix identified three additional epitopes in Cll that are likely to be immunogenic (ranked 1, 2, and 3). One was previously reported in IEDB but not confirmed in HLA transgenic mice nor in human T cell assays.

#### Malaria LSA

Finally, the single LSA epitope identified by both the Immunoinformatics approach and the CFS method contains a strong EpiMatrix-scoring peptide, and was the only LSA epitope to be validated in T cell assays. The weaker LSA epitope (C-LSA11) was also identified by the immunoinformatics approach, but the EpiMatrix score was slightly below the normal cut off for selection. Two additional epitopes, I-LSA1 and I-LSA3 were identified using EpiMatrix, of which one (I-LSA1) is found in IEDB for an unreported Class II HLA allele.

## Discussion

The CFS method described by Hartman et al. is a novel approach for identification of T cell epitopes that involves a significant amount of effort, reagents, and highly technical expertise in the use of MALDI mass-spectrometry. One advantage of CFS is that the method could, in theory, be applied to additional antigens and alleles for which immunoinformatics approaches do not yet exist. In comparison to the immunoinformatics approach, EpiMatrix, it appears to be both time- and cost-intensive. Many of the same epitopes are identified using the two approaches. Additionally, previously validated epitopes are identified using EpiMatrix that appear to be missed by the CFS (when comparing CFS and EpiMatrix results with the IEDB database of published and validated epitopes), as are several epitopes that appear likely to be immunogenic based on their rank and EpiMatrix score. Thus antigen-processing as performed using the CFS may have destroyed peptides that might otherwise have been presented by DR1. Two epitopes were identified using the CFS that would not have been selected by EpiMatrix. Neither of these epitopes would qualify as high quality targets based on information obtained from IEDB.

Epitopes identified using the CFS were not compared to epitopes that have already been published. Instead, in vivo methods were used to validate sequences. Epitopes were considered to be validated if, following immunization of HLA-DRB1*0101 mice with whole antigen, T cells were induced to proliferate, cytokine production was increased, or tetramer-stained cells could be detected, or some combination of the three. These methods are useful for validation regardless of the epitope selection method – and have been used for the validation of T cell epitopes identified using EpiMatrix (see references 6 and 9).

The CFS may provide some additional information on epitopes that are processed (two were identified in the CFS that were not identified using EpiMatrix) and may have application to organisms with little to no information on peptide-MHC interactions. A much more extensive (and perhaps cost-prohibitive) comparison would have to be performed, prospectively validating the epitopes discovered using EpiMatrix alongside epitopes discovered using the CFS, before either method is proven to be superior to the other. At the very least, they appear to be somewhat complementary.

It should be mentioned that there are a range of epitope prediction tools available, however each tool may provide slightly different results [3]. For example, when analyzing the sequences from H5N1 Vietnam and rLSA-1, both of which contain CFS-only epitopes, (Figure 1) with NetMHCII [10], an alternative epitope prediction tool, NetMHCII predicted the H5N1 Vietnam epitope but the rLSA-1 epitope was still not predicted. NetMHCII predicted similar epitopes to EpiMatrix for the proteins included in this analysis (data not shown) with the best scoring EpiMatrix epitopes centered within the sequences all of the strong binders predicted by NetMHCII. NetMHCII does not further delineate the optimal epitope when using its default peptide length of 15 amino acids (http://www.cbs.dtu.dk/services/NetMHCII/).

Immunoinformatics provides a rapid means of analyzing protein sequences for multiple HLA alleles at the same time. EpiMatrix prospectively identified more HLA-DRB1*0101 epitopes than the CFS, of which almost all are present in IEDB. In addition to providing an analysis of multiple alleles at once (not just HLA DRB1*0101) and highlighting promiscuous epitopes (data not shown, references 2 and 3 provide examples), the immunoinformatics approach can be performed at a fraction of the time and cost (one day, no reagents, versus multiple weeks, technician time, *in vitro* reagents, and MALDI mass spectrometer costs). Thus immunoinformatics approaches with algorithms such as EpiMatrix provide a clear cost and time advantage over CFS.

As the number of proteins that are being evaluated for putative immunogenicity expands, rapid, inexpensive and accurate tools are in great demand. The time and effort involved in prospectively identifying peptides from a single protein for a single allele were not reported for this study. Based on rough estimates for the costs, the immunoinformatics method can be performed in less than 1/20th of the time and for 1/100th of the cost of the cell free method. If one is to compare the cost of equipment that would be necessary for these two methods (a single internet accessible computer vs. a fully functional lab with mass spectrometry and protein production capabilities), this gap grows exponentially wider.

## Conclusions

Adding antigen-processing to epitope selection in vitro does not improve prospective class II antigen identification. In this comparison of two methods for T cell epitope identification, the in vitro antigen-processing and MHC-elution method (CFS) obscured two of three externally validated, HLA-DRB1*0101-restricted epitopes, whereas the immunoinformatics approach (EpiMatrix) correctly identified all published epitopes restricted by DRB1*0101, and prospectively identified many additional epitopes that have yet to be validated. While the CFS approach may enable identification of epitopes from MHC for which there is little to no published information, the advantages of the immunoinformatics method, which include a very high degree of accuracy, high throughput, rapidity, and low cost, are clear.

## Methods

### Immunoinformatics

Proteins analyzed by EpiMatrix are parsed into 9-mers that overlap each other by 8 amino acids. Each of these 9-mers is then scored individually (and simultaneously) for predicted binding affinity against a panel of eight common Class II HLA alleles (DRB1*0101, *0301, *0401, *0701, *0801, *1101, *1301, and *1501) that collectively cover over 95% of the human population [11]. EpiMatrix scores range from approximately −3 to +3. Those 9-mer peptides that score 1.64 or above are considered to be potential epitopes. The predictions have been benchmarked against the ‘gold standard’ set of published epitopes, performing as well or better than other epitope mapping tools [3]. Typically, 5% of all nine-mer frames in a given protein score above 1.64; these are considered to have a significant chance of binding to HLA molecules with moderate to high affinity and to have a significant chance of being presented on the surface of APCs [3].

## Figures and Tables

**Table 1:** Epitope Comparison Summary of epitopes identified by EpiMatrix and the CFS. In the case of I/C-PR1, I/C-Tex1, and C-VN45, multiple overlapping peptides were eluted in the CFS; those peptides sharing the same core 9-mer identified by EpiMatrix are considered one epitope. Variable flanking resides are indicated in grey text. Refer to Figure 1 for nomenclature and annotation.

Code (Fig. 1)	AA Sequence	Core EpiMatrix Epitope	DRB1*0101 Z-Score	IEDB Reference ID
C-VN45	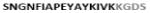	IAPEYAYKI	1.53	1009685**
C-LSA11	YDNFQDEENIGIYK	FQDEENIGI	1.58	1002410**
I/C-CII5	QTGEPGIAGFKGEQGPKGEPGPAGVQGAPGPAG	FKGEQGPKG	2.66	1007108*
I/C-PR1	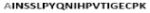	YQNIHPVTI	2.72	1000157^†^
I/C-Tex1	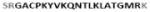	YVKQNTLKL	3.06	1000083*
I/C-LSA2	EDITFMKLGGSGSPHHHH	FMKLGGSGS	2.62	1002410**
I-CII1	YRSQKTSRL	YRSQKTSRL	3.14	N/A
I-CII2	FLRLLSTEG	FLRLLSTEG	2.98	N/A
I-CII3	FTGLQGLPG	FTGLQGLPG	2.75	1014795**
I-PR2	YQNENAYVS	YQNENAYVS	2.54	1000157^†^
I-PR3	WTLLKPGDT	WTLLKPGDT	2.53	1000157^†^
I-VN1	FHNIHPLTI	FHNIHPLTI	2.76	1018856**
I-VN2	LKHLLSRIN	LKHLLSRIN	2.74	1018856**
I-VN3	YIVEKANPV	YIVEKANPV	2.64	1009685*
I-LSA1	FKSLLRNLG	FKSLLRNLG	3.09	1002322**
I-LSA3	IKSNLRSGS	IKSNLRSGS	2.03	N/A
